# Ultrasound follow-up of spontaneous tears of the plantar fascia treated with conservative therapies: Two case reports

**DOI:** 10.1097/MD.0000000000018428

**Published:** 2019-12-27

**Authors:** Giulio Cocco, Vincenzo Ricci, Andrea Boccatonda, Michele Abate, Maria Teresa Guagnano, Cosima Schiavone

**Affiliations:** a Unit of Ultrasound in Internal Medicine, Department of Medicine and Science of Aging, “G. d’Annunzio” University, Chieti; b IRCCS Rizzoli Orthopaedic Institute, Department of Biomedical and Neuromotor Science, Physical and Rehabilitation Medicine Unit, Bologna, Italy.

**Keywords:** conservative therapy, follow-up, imaging, plantar fascia tear, ultrasound

## Abstract

**Rationale:**

The plantar fascia (PF) is an important anatomical structure that stabilizes the longitudinal arch of the foot. While plantar fasciitis is a common pathology affecting the foot, tears of the PF are uncommon injuries characterized by acute pain in the plantar aspect of the foot. The main purpose of this paper was to describe, in detail, how the ultrasonographic pattern of PF rupture can be combined with the clinical features to define the prognosis and promptly plan the therapeutic approach.

**Patient concerns:**

In the first case, a 39-year-old male patient was seen due to acute pain in the mid plantar foot which appeared 3 days after a tennis match. The pain was accompanied by a “snap” noise and intense pain. In the second case, a 44-year-old male patient was seen due to pain in the heel region which appeared 2 days after a running session.

**Diagnosis:**

One case of noninsertional complete tear of the central bundle of the PF with retraction of the 2 stumps and 1 case of partial tear of the central bundle of the PF at the level of the insertional region.

**Interventions:**

Both patients were treated with conservative therapies including load management, oral nonsteroidal anti-inflammatory drugs, foot orthosis, and restriction of sport activities.

**Outcomes:**

At follow-up, the patient with spontaneous complete tear of the PF (noninsertional area) showed a small fibrous bridge between the 2 stumps, with partial alignment of the proximal and distal portions, the ability to walk for a medium to long-distance, and difficulty going up and downstairs. The patient with the spontaneous partial tear (insertional area) showed complete fibrous scar tissue with restoration of the fascial continuity, and the ability to walk for a long-distance and go up and downstairs without pain.

**Lessons:**

Based on the clinical and ultrasonographic findings, we suggest that partial tear of the PF in the insertional region presents a favorable prognosis with complete recovery, both clinically and anatomically, while a complete tear in the noninsertional region is associated with partial functional and histological recovery when managed with a conservative approach. Therefore, coupling the clinical findings with the sonohistologic pattern is a valuable approach to plan the most suitable treatment for patients with spontaneous PF tear.

## 1 Introduction

The plantar fascia (PF) (PF; plantar aponeurosis) is an important anatomical structure that stabilizes the longitudinal arch of the foot. It consists of 3 main bundles: medial, central, and lateral. The central bundle is the thickest one, starting from the medial tuberculum of the calcaneus and extending forward to cover the plantar surface of the flexor digitorum brevis muscle. The lateral bundle beneath the plantar surface of the abductor digiti quinti muscle continues laterally with the dorsal fascia and distally with the fifth metatarsophalangeal joint capsule. The medial bundle of the PF is the thinnest one, located under the plantar surface of the abductor hallucix muscle, extending proximally to the flexor retinaculum of the foot and distally to the first metatarsophalangeal joint capsule.[^1^,^2^]

Plantar fasciitis is commonplace in daily clinical practice, and is estimated to be responsible for more than 1 million patients seeking treatment annually.^[3]^ Despite the name of the pathology, which suggests an inflammatory etiology, it actually presents a degenerative nature related to overuse and/or trauma, with disorganization of the tissue architecture and microtears.^[4]^ It is a multifactorial disorder comprised of biomechanical causes (pathological gait), foot deformities (flatfoot), improper footwear, overweight and rheumatic diseases (eg, seronegative spondyloarthropathies and rheumatoid arthritis).[^5^,^6^] While plantar fasciitis is a common foot pathology, PF rupture, first described in 1978 by Leach et al,^[7]^ is not common. The proximal third of the central bundle of the PF is usually involved in degenerative conditions caused by overuse and microtraumas, which can progress to partial or complete tears.^[4]^ In clinical practice, the extension of the injury (partial/complete), the exact anatomical location (insertional/noninsertional) and the etiology of the damage (traumatic/atraumatic) are all fundamental for planning specific and prompt surgical or conservative management.

Traumatic tears are often related to forcible plantar flexion of the foot (eg, in competitive athletes such as runners and jumpers), and typically occur distal to the calcaneal insertion of the PF. Thus, chronic overuse is usually considered an aetiological factor in these patients.[^8^,^9^] Spontaneous ruptures may occur at the calcaneal attachment of the PF in patients with a previous history of plantar fasciitis and local treatment with corticosteroid injection(s).[^10^,^11^,^12^] The typical clinical presentation of PF injuries involves acute pain, a “snap” noise and local swelling.^[11]^

## 2 Case descriptions

Here, we report 2 cases of spontaneous tear of the PF in patients with no preceding symptoms or steroid injections. The clinical findings were confirmed by diagnostic ultrasound (US) examination, which revealed severe fasciopathy with structural lesions.

### 2.1 Case 1 (complete tear)

**Figure 1:**
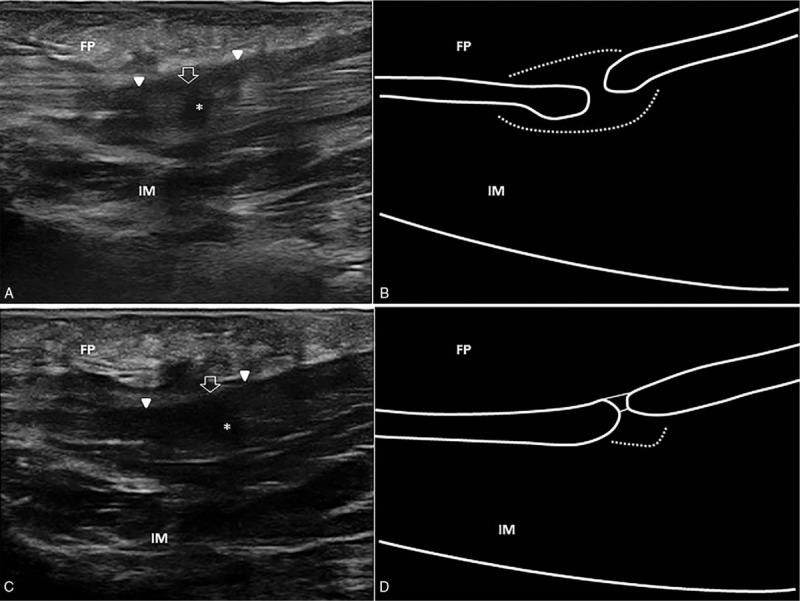
Complete tear. Acute phase. Ultrasound image showing complete tear of the mid-portion of the plantar fascia (white void arrow), with retraction of the 2 stumps (white arrowheads) and local effusion (asterisk) (A). Corresponding schematic drawing of the complete plantar fascia lesion with the gap-sign and perilesional edema (white dotted line) (B). Follow-up at 2 months. Ultrasound image shows a small fibrous bridge (white void arrow) between the 2 stumps (white arrowheads) and reduction of the local edema (C). Corresponding schematic drawing showing the alignment of the proximal and distal segments of the plantar fascia with a small amount of perilesional edema (white dotted line) (D). FP = fat pad, IM = intrinsic muscles.

A 39-year-old male patient was seen due to acute pain in the mid plantar foot which appeared 3 days after a tennis match. The pain was accompanied by a “snap” noise and intense pain. Physical examination revealed local swelling, ecchymosis, and tenderness at the level of the heel and plantar foot, in addition to painful dorsiflexion of the first metatarsophalangeal joint. US examination was performed in accordance with the standard procedure for scanning the plantar region of the foot,^[13]^ which showed a complete tear of the central bundle of the PF with retraction of the 2 stumps and perilesional edema (Fig. 1A and B). The patient was treated conservatively with oral nonsteroidal anti-inflammatory drugs for the first 5 days to reduce the local edema, then switched to acetaminophen as needed to control the pain. No load-bearing was allowed for the first 2 weeks after the injury (walking with crutches), then partial load-bearing was performed for the following 7 days. Insoles to support the longitudinal arch of the foot were prescribed, and sport activities were avoided for 5 weeks. Two months later, US examination showed a remarkable reduction in local soft tissue edema with a small fibrous bridge between the 2 stumps and acceptable alignment of the proximal and distal segments of the PF (Fig. 1C and D). At follow-up, the patient was able to walk for a medium to long-distance using a footwear with a rear rise and insole, but walking up and downstairs was still painful.

### 2.2 Case 2 (partial tear)

**Figure 2:**
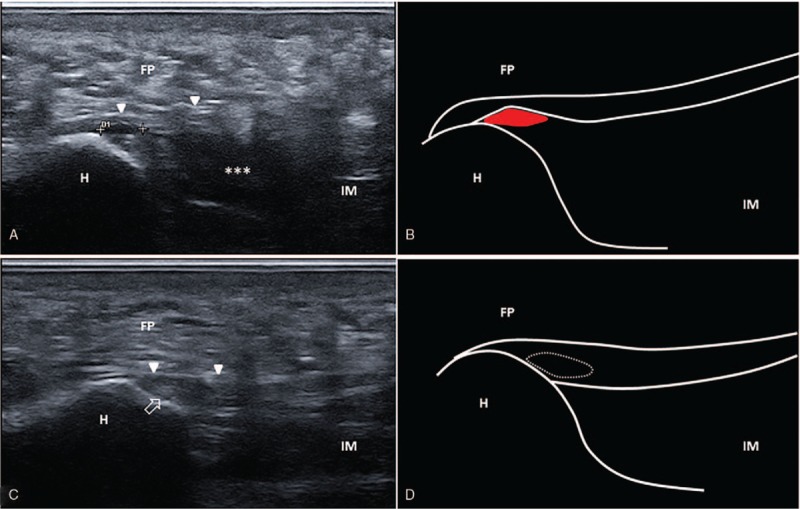
Partial tear. Acute phase. Ultrasound image showing partial tear of the plantar fascia (double cross) at the level of the insertional region over the calcaneal tuberosity, with post-traumatic edema of the intrinsic muscles of the foot (asterisks) and inhomogeneous fat pad (A). Corresponding schematic drawing of the partial plantar fascia lesion (red) (B). Follow-up at 2 months. Ultrasound image shows the fibrous scar tissue (white void arrow) at the level of the insertional region with a correct restoration of the fascial continuity (white arrowheads) (C). Corresponding schematic drawing of the plantar fascia scar (white dotted line) (D). White arrowheads indicate the plantar fascia. FP = fat pad, H = heel, IM = intrinsic muscles.

A 44-year-old male patient was seen due to pain in the heel region which appeared 2 days after a running session. Physical examination revealed local swelling at the level of the calcaneal region and plantar foot and moderate pain during dorsiflexion of the first metatarsophalangeal joint. US examination was performed in accordance with the standard procedure for scanning the plantar region of the foot,^[13]^ which showed a partial tear of the central bundle of the PF at the level of the insertional region over the medial calcaneal tuberosity (Fig. 2A and B). The patient was treated conservatively with nonsteroidal anti-inflammatory drugs or acetaminophen as needed to control the local pain. Partial load on the lower affected limb (using crutches) was prescribed for 2 weeks after the injury, and insoles to support the longitudinal arch of the foot were prescribed with rocker bottom shoes to limit rolling. Sport activity was avoided for 4 weeks. Two months later, US examination revealed a fibrous scar tissue with correct restoration of the fascial continuity at the insertional region, in addition to remarkable reduction of edema in the heel fat pad and intrinsic muscles of the plantar foot (Fig. 2C and D). At follow-up, the patient was able to walk for a long-distance and walk up and downstairs without pain.

## 3 Discussion

The diagnosis of PF tear mainly relies on the patient's medical history and on physical examination. A history of acute pain in the sole of the foot during activity, together with clinical findings of a tender lump, local swelling from subcutaneous bleeding and often accompanied by a “snap” noise, suggest the diagnosis.^[11]^ However, instrumental investigations, such as US and magnetic resonance imaging (MRI), allow the physician to perform differential diagnosis and determine the severity of the injury, which are not possible with physical examination alone.^[13]^

US examination is the first-line modality to localize the tissue lesion and measure its extension. Furthermore, dynamic maneuvres facilitate confirmation of a complete tear by verifying the presence of a gap between the 2 torn parts of the PF.^[14]^ Sonography is a repeatable (follow-up of the patient), cost-effective and time-saving imaging modality that allows for dynamic evaluation and comparison with the contralateral side. Moreover, US imaging is superior to MRI in differentiating true fiber interruption from local edema, especially during the acute or sub-acute phase.^[15]^ However, in cases with a complete tear, precise information on PF retraction for an eventual surgical repair is best obtained with MRI.^[15]^ We reported 2 cases of PF tear in healthy subjects with no specific risk factors (ie, plantar fasciitis or local steroid injections), with clinical and ultrasonographic follow-up at 2 months. For the spontaneous complete tear of the PF (noninsertional area), a small fibrous bridge between the 2 stumps with partial alignment of the proximal and distal portions was observed at follow-up; whereas for the spontaneous partial tear (insertional area), complete fibrous scar tissue with restoration of the fascial continuity was observed at 2 months.

Based on the clinical and ultrasonographic findings, we speculate that a partial tear of the PF in the insertional region could be considered a lesion with a favorable prognosis which is expected to recover completely, both clinically and anatomically. Complete restoration of the fascial continuity in the insertional area is probably related to the continuous microtraumas that occur during walking, and; therefore, greater stimulation by local growth factors. On the other hand, complete tear of the PF in the noninsertional region could be considered a lesion with partial functional and histological recovery when managed with a conservative approach. Acceptable alignment of the 2 stumps and an incomplete connection of the proximal and distal segment of the PF with a fibrous bridge could be sufficient for daily activities but not for sport activities. The gap between the 2 stumps and the distance from the loading zones of the foot (ie, poor stimulation by mechanical forces and local growth factors) are probably correlated with poor clinical and ultrasonographic outcomes.

## Author contributions

**Conceptualization:** Giulio Cocco.

**Data curation:** Giulio Cocco.

**Investigation:** Giulio Cocco, Vincenzo Ricci, Maria Teresa Guagnano.

**Methodology:** Andrea Boccatonda.

**Project administration:** Vincenzo Ricci.

**Supervision:** Michele Abate, Cosima Schiavone.

**Validation:** Andrea Boccatonda, Cosima Schiavone.

**Writing – original draft:** Giulio Cocco.

**Writing – review and editing:** Vincenzo Ricci, Andrea Boccatonda, Cosima Schiavone.
